# Effects of Cerebral Blood Flow and White Matter Integrity on Cognition in CADASIL Patients

**DOI:** 10.3389/fpsyt.2018.00741

**Published:** 2019-01-14

**Authors:** Xinzhen Yin, Ying Zhou, Shenqiang Yan, Min Lou

**Affiliations:** Department of Neurology, School of Medicine, The Second Affiliated Hospital of Zhejiang University, Hangzhou, China

**Keywords:** *NOTCH3*, CADASIL, diffusion tensor imaging, cognitive impairment, cerebral blood flow, arterial spin labeling

## Abstract

**Background:** It remains unclear whether the degree of white matter tract damage or cerebral hypoperfusion can better predict global cognitive impairment in CADASIL. We sought to determine the independent effects of cerebral perfusion status and white matter integrity on the cognition.

**Methods:** We reviewed prospectively collected clinical and imaging data from genetically-confirmed CADASIL patients who underwent both arterial spin labeling (ASL) perfusion MRI and diffusion tensor imaging (DTI). We analyzed the cerebral blood flow (CBF), mean diffusion (MD), and fractional anisotropy (FA) by dividing the brain tissue into white matter hyperintensity (WMH) and normal-appearing white matter (NAWM). Global cognitive function was evaluated by using Mini-Mental State Examination (MMSE) and the Montreal Cognitive Assessment (MoCA).

**Results:** Of the included 29 CADASIL patients, the mean age was 48.4 ± 7.9 years, and 17 (58.6%) were women. MD was significantly correlated with CBF in both WMH (*r* = −0.407, *P* = 0.035) and NAWM (*r* = −0.437, *P* = 0.023) after adjusting for age and WMH volume. A MoCA score was obtained in 13 patients and was significantly correlated with CBF in both WMH (*r* = 0.742, *P* = 0.004) and NAWM (*r* = 0.659, *P* = 0.014). Both CBF in WMH (area under the curve, 0.767; 95% CI, 0.586-0.947, *P* = 0.015) and MD in WMH (area under the curve, 0.740; 95% CI, 0.557–0.924, *P* = 0.028) were good predictors for cognitive impairment (MMSE score < 27). However, multiple linear regression analysis revealed that global cognitive function was independently associated with CBF in WMH only (standardized β = 0.485, *P* = 0.015), after adjusting for age, gender, WMH volume, the presence of subcortical infarcts and DTI metrics.

**Conclusions:** Our findings suggested that cerebral hypoperfusion was more strongly associated with global cognitive dysfunction than the severity of brain microstructural damage, supporting that CBF assessed by ASL could serve as a candidate imaging indicator for monitoring alterations of global cognitive function in CADASIL.

## Introduction

Cerebral autosomal dominant arteriopathy with subcortical infarcts and leukoencephalopathy (CADASIL) is an early-onset monogenic variant of cerebral small vessel disease (CSVD) caused by mutations in the *NOTCH3* gene (1), whose prevalence is at least 4.6 per 100,000 adults (2). Pathologically, there is deposition of granular osmiophilic material in the basal membrane of small arteries and capillaries in close association with progressive degeneration of smooth muscle cells (3, 4). Cognitive impairment is the second most frequent clinical manifestation of CADASIL, principally affecting processing speed, executive function, and attention from an early stage (5–7).

The mechanism of cognitive dysfunction in CADASIL remains uncertain. No strong correlation has been found between cognitive function and T2 lesion load on conventional MRI (8), since T2 high signal can be caused by severe neuronal loss or subtle damage to vascular tissues that leaves neural fibers intact. Therefore, diffusion tensor imaging (DTI), which is sensitive to the microstructural integrity of white matter, is used in the CADASIL population. Previous studies have shown that the degree of white matter tract damage may relate to global cognitive function (9, 10). On the other hand, cerebral hypoperfusion was also found to be correlated with cognitive impairment or dementia in CADASIL (11, 12). However, interaction may exist between cerebral hypoperfusion and disruption of brain microstructure. It's unclear whether white matter integrity or cerebral perfusion condition could better predict the global cognitive outcome.

To our knowledge, few previous studies have investigated the relationship between cerebral perfusion status and white matter integrity or their correlations with cognitive function in CADASIL. We thus performed both 3D arterial spin labeling (ASL) perfusion MRI, which provides measures of cerebral blood flow (CBF), and DTI in genetically-confirmed CADASIL patients, in order to determine the independent effects of CBF and DTI metrics on cognition.

## Materials and Methods

### Study Subjects

This was an investigator-initiated prospective single-center study. During the years 2007–2017, we performed *NOTCH3* gene testing in patients with probable CADASIL. CADASIL suspicion arose when typical clinical features (migraine, stroke, cognitive deficits, or psychiatric symptoms), positive family history, or neuroimaging were suggestive of an inherited CSVD. Affected family members of index patients were not included in the current study. We then enrolled patients who (i) had a deleterious mutation of *NOTCH3*; (ii) underwent both ASL and DTI at the same time; and (iii) received a cognitive function assessment based on the Mini-Mental State Examination (MMSE) and/or the Montreal Cognitive Assessment (MoCA). We excluded patients whose image quality was poor due to motion artifacts. This study has been approved by our local human ethics committee. All clinical investigation has been conducted according to the principles expressed in the Declaration of Helsinki. Informed consent was obtained for all patients.

We retrieved demographic, clinical, and radiological data including age (disease onset and first visit) and gender; the vascular risk factors such as history of hypertension, diabetes mellitus, hyperlipidemia, and smoking; clinical features including ischemic events, migraine, family history, MMSE and MoCA score; and conventional neuroimaging findings such as intracranial arterial stenosis, the severity of white matter hyperintensities (WMHs), temporal poles hyperintensity, external capsule involvement, and subcortical infarcts (single and multiple) were recorded. Family history was collected by means of a structured interview that focused on the typical CADASIL disturbances referred to by all the proband's relatives. The family history was considered positive when at least one typical disturbance was present in at least one of the proband's first-degree relatives.

### MRI Parameters

MRI was performed on a 3.0T system (MR750, GE Healthcare, United States) equipped with an 8-channel phased array head coil. MR sequences contained high-resolution 3D sagittal T1-weighted imaging (T1-WI), fluid attenuated inversion recovery (FLAIR), DTI and 3D ASL. A single shot, diffusion-weighted spin echo echo-planar imaging sequence was performed for DTI. Maximum b-value was 1,000 s/mm^2^ in 30 non-collinear directions; one volume was acquired without diffusion weighting (b-value = 0 s/mm^2^). Other parameters of DTI were as follows: TR = 8,000 ms; TE = 80.8 ms; flip angle = 90°; FOV = 25.6 × 25.6 cm^2^; matrix size = 128 × 128; slice thickness = 2.0 mm without interslice gap. 3D ASL was acquired using spin-echo pulse sequence with TR/TE = 4611/10.5 ms, TI = 1,525 ms, flip angle = 111°, slice thickness = 4 mm, matrix = 128 × 128, FOV = 24 × 24 cm^2^. High-resolution 3D sagittal T1WI was acquired using spoiled gradient echo sequence with TR/TE = 7.3/3.0 ms, TI = 450 ms, flip angle = 8°, slice thickness = 1 mm, matrix = 250 × 250, FOV = 25 × 25 cm^2^. Time-of-flight magnetic resonance angiography (TOF-MRA) consisted of 3 slabs with TR = 20 ms; TE = 3.2 ms; flip angle = 15°; FOV = 24 × 24 cm^2^; matrix size = 320 × 224; slice thickness = 1.4 mm. FLAIR parameters were TR = 9,000 ms; TE = 150 ms; TI = 2,250 ms; FOV = 24 × 24 cm^2^; matrix size = 256 × 192; slice thickness = 5.0 mm. Axial FLAIR sequence was used to measure the lesion volume of WMHs with the following parameters: TR = 8,400 ms; TE = 152 ms; FOV = 24 × 24 cm^2^ matrix size = 256 × 256; flip angle = 90°; TI = 2,100 ms; slice thickness = 4.0 mm without interslice gap. The whole brain was imaged.

### *NOTCH3* Gene Analysis

We used the diagnostic strategy established by Joutel et al. (13). We initially screened exons 3 and 4 for mutations, and if no mutations were present, we then analyzed the remaining exons, i.e., 2 and 5–23. Co-segregation was analyzed if any variant was found, and the presence of all identified, novel, disease-associated variants was examined in 100 controls by direct DNA sequencing.

### Imaging Analysis

**Figure 1:**
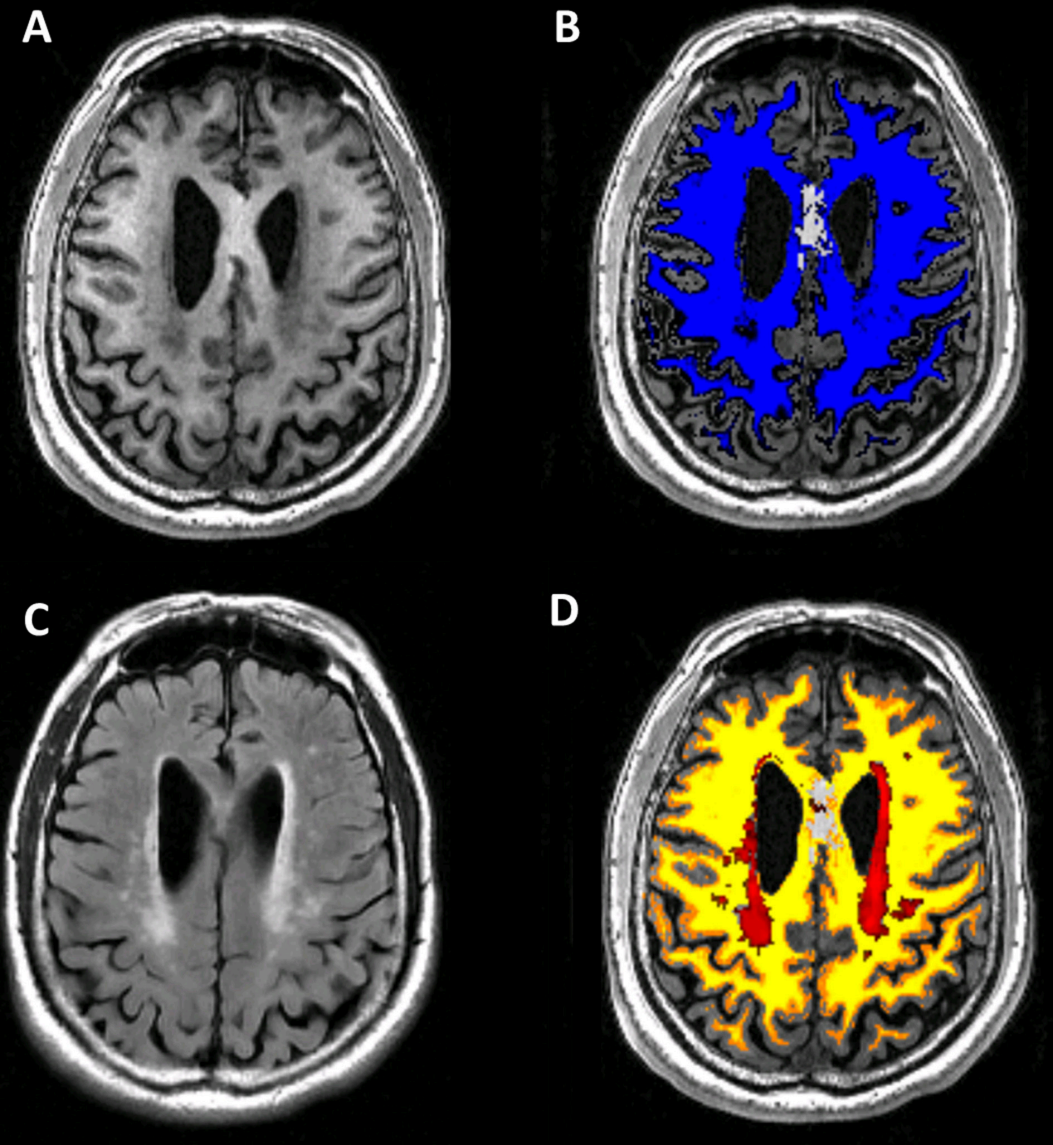
Flowchart demonstrating our imaging process for each subject. The 3DT1 image **(A)** was segmented to white mater mapping **(B)** automatically on Statistical Parametric Mapping (SPM). Then the T2-FLAIR image **(C)** was co-registered to 3DT1 image. White matter hyperintensity (WMH) lesion segmentation was based on co-registered T2FLAIR image **(C)** on Lesion Segmentation Tool in SPM. Normal-appearing white mater mapping **(D)** was obtained by image subtraction of white mater mapping and WMH lesion.

DTI images were post-processed using FSL (www.fmrib.ox.ac.uk/fsl) to extract brain, remove bulk motion, and eddy current induced distortions. Then we calculated the parametric maps of mean diffusivity (MD) (a measure of the apparent diffusion coefficient averaged in all spatial directions), and fractional anisotropy (FA) (a measure of the directionality of diffusion) with DTIfit command in FSL. The raw data of ASL were transferred to a separate workstation (ADW, GE), where the quantitative CBF maps were generated by a custom-built program. The segmentation of normal-appearing white matter (NAWM) and WMH tissue masks was automatically processed in the native space using 3D T1WI and FLAIR images by the lesion segmentation tool (LST) toolbox in Statistical Parametric Mapping Version 8 (SPM8) (Figure 1) (14). The processed WMH and NAWM masks were further manually corrected by using ITK-SANP software (www.itksnap.org). The steps of manual correction included (i) removal of non-brain tissue, deep gray matter, brain stem, and cerebellum; and (ii) correction of false segmentation (positives or negatives). After co-registration, the masks of WMH and NAWM were used to obtain averaged MD, FA, and CBF of corresponding tissues in each subject.

### Statistical Analysis

All numeric variables were expressed as mean ± SD. The difference between FA, MD, and CBF in WMH and their counterparts in NAWM were compared using paired *t*-tests. Pearson's correlation analysis was used for the continuous variables. In addition, we performed partial Pearson's correlation analysis to determine the correlation between CBF and the metrics of DTI (FA and MD) by adjusting for age and WMH volume. The association of CBF, MD, FA, age, gender, WMH volume, and the presence of subcortical infarcts was tested using the univariate linear regression model. The association of the variables, whose *P* < 0.1, with MMSE score was estimated using the multiple linear regression model. Receiver operating characteristic curve analysis was used to determine predictive value. All analyses were performed blinded to participant identifying information. Statistical significance was set at a *P* < 0.05. All statistical analyses were performed with SPSS package.

## Results

A total of 29 genetically-confirmed CADASIL patients were included for the final analysis. The demographic, clinical and imaging characteristics were demonstrated in Table 1. The age at disease onset was 42.0 ± 9.4 years (ranged from 21 to 57 years), and the age at first neurological examination was 48.4 ± 7.9 years (ranged from 31 to 63 years), and 17 (58.6%) were women. The MMSE score was 23.8 ± 6.9 (ranged from 7 to 30). Seventeen (58.6%) of them suffered at least one transient ischemic attack or completed stroke, 10 (34.5%) had a migraine with aura, and 25 (86.2%) had a positive family history. Only one patient had severe arterial stenosis (right middle cerebral artery), with a MMSE score of 27 and MoCA score of 15. No obvious intracranial arterial stenosis was shown in the other patients.

**Table 1:** Main characteristics of CADASIL patients.

**Variable**	***N* = 29 (mean ± SD) or *n* (%)**
Age at disease onset (year)	42.0 ± 9.4
Age at first neurological examination (year)	48.4 ± 7.9
Female	17 (58.6%)
**Clinical features**	
Ischemic TIA/stroke	17 (58.6%)
MMSE score	23.8 ± 6.9
Migraine with aura	10 (34.5%)
Family history	25 (86.2%)
**Vascular risk factors**	
Hypertension	4 (13.8%)
Diabetes	1 (3.4%)
Hyperlipidemia	6 (20.7%)
Smoking	6 (20.7%)
**Neuroimaging findings**	
Lesion load of WMH (ml)	64.1 ± 31.7
Temporal poles hyperintensity	18 (62.1%)
External capsule involvement	21 (72.4%)
Subcortical infarcts	20 (69.0%)
Single	3 (15.0%)
Multiple	17 (85.0%)

*CADASIL, cerebral autosomal dominant arteriopathy with subcortical infarcts and leukoencephalopathy; SD, standard deviation; TIA, transient ischemic attack; MMSE, Mini-mental state examination; WMH, white matter hyperintensity*.

**Figure 2:**
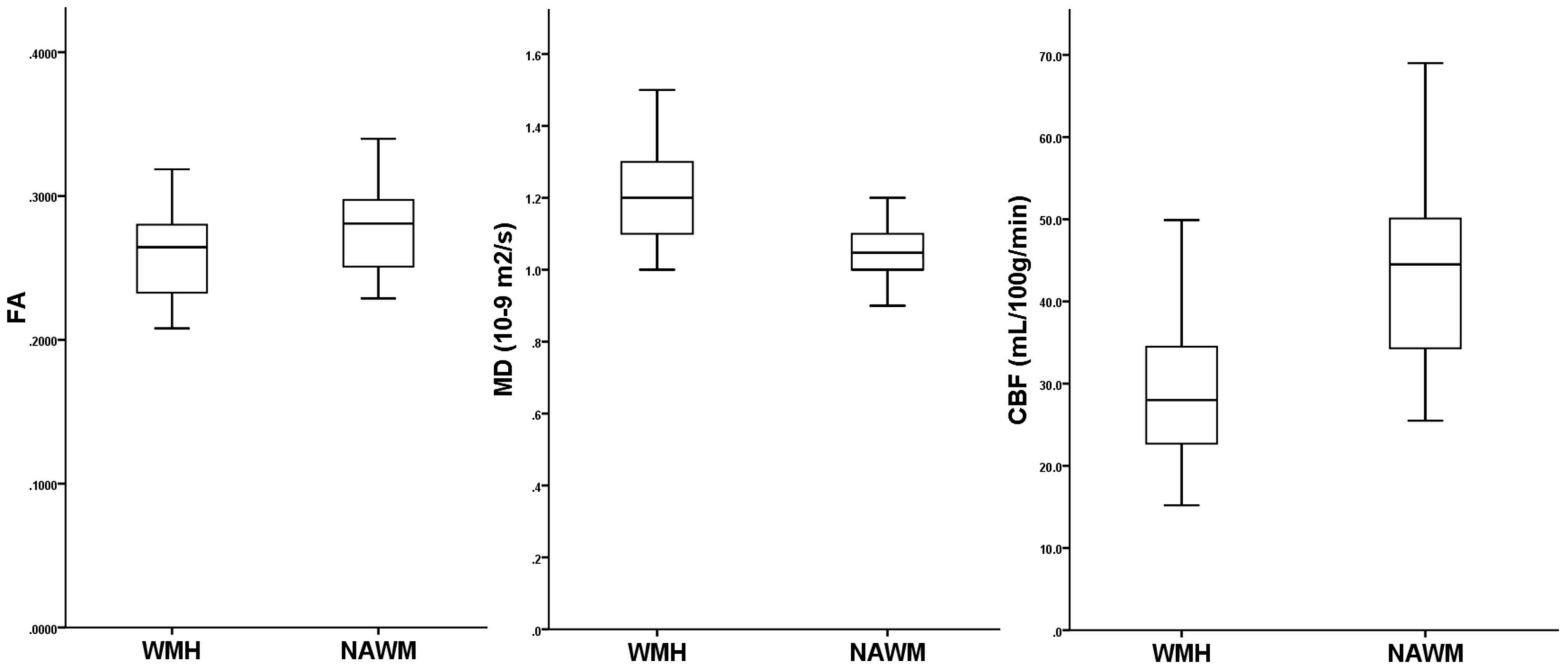
Comparisons of fractional anisotropy (FA), mean diffusion (MD) and cerebral blood flow (CBF) in white matter hyperintensities (WMH) and their corresponding normal-appearing white matter (NAWM).

MD, FA, and CBF in WMH and NAWM were not associated with age (all *P* > 0.05). There existed a significant association between the WMH volume and FA in WMH (Pearson *r* = −0.543, *P* = 0.002), and in NAWM (Pearson *r* = −0.757, *P* < 0.001), while a tendency was detected with MD in WMH (Pearson *r* = 0.322, *P* = 0.088) and in NAWM (Pearson *r* = 0.363, *P* = 0.053). As illustrated in Figure 2, FA and CBF were significantly decreased in WMH compared to NAWM, while MD was significantly increased. After adjusting for age and WMH volume, MD was significantly correlated with CBF in both WMH (*r* = −0.407, *P* = 0.035) and NAWM (*r* = −0.437, *P* = 0.023), while there lacked of an association between FA and CBF in both WMH (*r* = 0.196, *P* = 0.328) and NAWM (*r* = 0.159, *P* = 0.427) (Table 2).

**Table 2:** Partial correlation between cerebral blood flow and DTI-derived indices after adjusting for age and lesion load of WMH.

	**CBF-WMH**		**CBF-NAWM**	
	***r***	***P* value**	***r***	***P* value**
FA-WMH	0.196	0.328	0.288	0.146
FA-NAWM	0.146	0.469	0.159	0.427
MD-WMH	**−0.407**	**0.035**	−0.380	0.051
MD-NAWM	−0.235	0.238	**−0.437**	**0.023**

*DTI, diffusion-tensor imaging; WMH, white matter hyperintensity; CBF, cerebral blood flow; NAWM, normal-appearing white matter; FA, fractional anisotropy; MD, mean diffusivity. Bold indicates p < 0.05*.

Univariate linear regression analysis demonstrated that MMSE score was significantly associated with CBF in both WMH and NAWM, and mean FA and MD in WMH, but not in NAWM (Table 3). Nevertheless, MMSE score was not significantly associated with WMH volume (standardized β = −0.189, *P* = 0.325). The cut-off point of CBF in WMH was 27 mL/100 g/min (area under the curve, 0.767; 95% CI, 0.586–0.947, *P* = 0.015), and this yielded a sensitivity of 73.3% and a specificity of 78.6% for prediction of cognitive impairment (MMSE score < 27), while CBF in NAWM was not a good predictor (area under the curve, 0.676; 95% CI, 0.480–0.872, *P* = 0.106). The cut-off point of MD in WMH was 1.250 10^−9^ m^2^/s (area under the curve, 0.740; 95% CI, 0.557–0.924, *P* = 0.028), and this yielded a sensitivity of 71.4% and a specificity of 60.0% for prediction of cognitive impairment, while FA in WMH was not a good predictor (area under the curve, 0.629; 95% CI, 0.419–0.838, *P* = 0.239). MoCA score was obtained in 13 patients. After adjusting for age and WMH volume, MoCA score was significantly correlated with CBF in both WMH (*r* = 0.742, *P* = 0.004) and NAWM (*r* = 0.659, *P* = 0.014), while there was a trend toward significance between MoCA and MD in WMH (*r* = −0.519, *P* = 0.069). Multiple linear regression analysis revealed that global cognitive function was independently associated with CBF in WMH only (standardized β = 0.485, *P* = 0.015), after adjusting for age, gender, WMH volume, the presence of subcortical infarcts, and DTI metrics (Table 3).

**Table 3:** Univariate and multivariate linear regression analyses of imaging variables with MMSE score.

	**Univariate**		**Multivariate**	
	**Standardized β**	***P* value**	**Standardized β**	***P* value**
FA-WMH	**0.378**	**0.043**	0.262	0.386
FA-NAWM	0.337	0.074	–	**–**
MD-WMH (10^−9^ m^2^/s)	**−0.409**	**0.028**	−0.133	0.602
MD-NAWM (10^−9^ m^2^/s)	−0.285	0.134	–	–
CBF-WMH (mL/100g/min)	**0.452**	**0.014**	**0.485**	**0.015**
CBF-NAWM (mL/100g/min)	**0.439**	**0.017**	0.205	0.565

*MMSE, Mini-mental state examination; FA, fractional anisotropy; WMH, white matter hyperintensity; NAWM, normal-appearing white matter; MD, mean diffusivity; CBF, cerebral blood flow. Age, gender, lesion load of WMH, and the presence of subcortical infarcts were adjusted in the multiple linear regression models. Bold indicates p < 0.05*.

## Discussion

In the current study, we investigated the effects of both cerebral perfusion status and white matter integrity on cognitive function in CADASIL patients. Our findings suggested that cerebral hypoperfusion was more strongly associated with global cognitive dysfunction than the severity of brain microstructural damage, which might differ from that in sporadic CSVD.

Although almost all demented CADASIL patients appeared to have confluent and diffuse WMH, some asymptomatic or mildly affected subjects had similar lesions (15). Therefore, the extent of WMH on T2-weighted images or FLAIR did not account for the phenotypic severity in CADASIL. Chabriat et al. first used DTI to detect the microstructural tissular alterations underlying T2 signal abnormalities and found that water diffusivity measured within WMH correlated with both MMSE and Rankin scale scores (9). Another DTI study also showed that the diffusion abnormalities of the thalamus correlated with cognitive function in CADASIL without dementia, especially for executive dysfunction (10). In addition, attentional network connectivity was proven to be associated with cognitive performance in CADASIL based on functional MRI (16), while the increased rapid-onset cortical plasticity might contribute to largely preserved cognitive function despite extensive ischemic changes (17).

Increased *Notch3* activity mediates reduction in maximal dilator capacity of cerebral arteries in CADASIL and may contribute to reductions in CBF (18). Previous studies also reported the relationship between cerebral hypoperfusion and cognitive dysfunction. A SPECT study of a German CADASIL family showed that cognitive impairment was linked to hypoperfusion in the basal ganglia, and demented patients had a pattern of frontal, temporal, and basal ganglial hypoperfusion (19). A significant reduction in absolute and relative CBF was found within areas of WMH, and this reduction was more severe in demented than in non-demented CADASIL patients (12). Another perfusion metric of cerebral blood volume was also proved to be correlated with disability and cognitive impairment in CADASIL (11).

In the current study, we found that both CBF and MD in WMH were good predictors for cognitive impairment according to receiver operating characteristic curve analysis, while multiple linear regression analysis revealed that CBF in WMH was more strongly associated with global cognitive function. There is no doubt that both cerebral hypoperfusion and brain microstructural damage contribute to cognitive decline. However, brain microstructural changes appear secondary to cerebral perfusion changes, considering both the association between regional MD and CBF, and the results of multiple linear regression analysis. Cerebral hypoperfusion in *NOTCH3* mutation carriers was supposed to precede the development of brain microstructural damage. CADASIL patients had both impaired cerebral and peripheral vasoreactivity at an early stage (20), and hemodynamic parameters were found to be abnormal in the superficial nerve fiber layer of the optic nerve head and retinal capillaries (21, 22). Chronic cerebral hypoperfusion reduced the activity of extracellular signal-regulated kinases, leading to neuronal adaptive responses, and impaired the function of microglial cells, which were implicated in amyloid-β elimination (23). Interestingly, both cognitive decline and cerebral hypoperfusion improved in a CADASIL patient during 2-year administration of lomerizine (24). Our findings suggested that CBF assessed by ASL could serve as a candidate imaging indicator for monitoring alterations of global cognitive function in CADASIL.

Our study had several limitations. First, although we prospectively collected data using a CADASIL registry and MRI protocol, our study design was cross-sectional. Longitudinal studies are needed to explore the causality between cerebral hypoperfusion, white matter integrity and cognitive outcome. Second, the number of CADASIL patients included in the current study was small, which reduced the power to detect significant effects and precluded comprehensive statistical analysis. Third, the MMSE is a crude measure of cognitive functioning that is insensitive to executive dysfunction and is not sensitive enough to detect mild cognitive impairment. Since only part of the enrolled subjects had a MoCA score, the multivariate analysis for MoCA was inapplicable due to the small sample size. More specific tests of executive function and neuropsychological assessment are required in future studies. Fourth, we did not focus on the CBF in subcortical gray matter nuclei, which might also contribute to the cognitive function. Moreover, the emerging technique of diffusion kurtosis imaging allows the measurement of mean kurtosis, which does not require tissue's directionality and hence it could provide more detailed information of microstructural integrity than DTI. The relationship between mean kurtosis and cognitive function should be further investigated in CADASIL.

In conclusion, our study demonstrated a significant association between cerebral hypoperfusion and the severity of brain microstructural damage, while the cerebral perfusion status was more strongly associated with global cognitive function than with white matter integrity.

## Author Contributions

XY drafted and revised the manuscript, participated in study concept and design, conducted the statistical analyses, analyzed and interpreted the data. SY participated in study concept and design, data interpretation and made a major contribution in revising the manuscript. ML participated in the study design and made contribution in revising the manuscript. YZ assisted in designing the MRI sequences and imaging analysis, and assessed the cognitive function of the participants.

### Conflict of Interest Statement

The authors declare that the research was conducted in the absence of any commercial or financial relationships that could be construed as a potential conflict of interest.
